# Characterization and Design Improvement of a Thickness-Shear Lead Zirconate Titanate Transducer for Low Frequency Ultrasonic Guided Wave Applications

**DOI:** 10.3390/s19081848

**Published:** 2019-04-18

**Authors:** Marco Zennaro, Dan J. O’Boy, Premesh Shehan Lowe, Tat-Hean Gan

**Affiliations:** 1 National Structural Integrity Research Centre, Granta Park, Great Abington, Cambridge CB21 6AL, UK; 2 Department of Aeronautical and Automotive, Loughborough University, Loughborough LE11 3TT, UK; d.j.oboy@lboro.ac.uk; 3 Brunel University London, Uxbridge UB8 3PH, UK; Shehan.lowe@brunel.ac.uk (P.S.L.); tat-hean.gan@brunel.ac.uk (T.-H.G.)

**Keywords:** ultrasonic guided waves testing, mode purity, non-destructive testing, numerical simulations, sensor development, thickness-shear transducers

## Abstract

Thickness-shear transducers for guided wave testing have been used in industry for over two decades and much research has been conducted to improve the resolution and sensitivity. Due to a geometric feature of the current state-of-the art transducer, there is an out-of-plane component in the propagation direction of the fundamental shear horizontal mode which complicates the signal interpretation. In such case, complex signal processing techniques need to be used for mode discrimination to assess the structural health with higher precision. Therefore, it is important to revise the transducer design to eliminate the out-of-plane components in the propagation direction of fundamental shear horizontal mode. This will enhance the mode purity of fundamental shear horizontal mode for its application in guided wave inspection. A numerical investigation has been conducted on a 3 mm thick 2 m circular steel plate to understand the behaviour and the characteristics of the state-of-the-art thickness-shear transducer. Based on the results, it is noted that the redesigning the electrode arrangement will suppress the out-of-plane components on the propagation direction of the fundamental shear horizontal mode. With the aid of this information current state-of-the-art transducers were redesigned and tested in laboratory conditions using the 3D Laser Doppler Vibrometer. This information will aid future transducer designers improve the resolution of thickness-shear transducers for guided wave applications and reduce the weight and cost of transducer array by eliminating the need of additional transducers to suppress spurious modes.

## 1. Introduction

For many decades, academic and industrial institutions have developed techniques in the field of Ultrasonic Guided Wave Testing (UGWT) as part of non-destructive inspection methods to assess the structural integrity of higher value engineering assets i.e., pipelines, rails and oil storage tanks to locate and determine the size of any potential mechanical defects. Any casualties in such assets caused by structural failures are generally catastrophic [1] or require greater levels of remanufacturing, costing resource and disruption. As a solution, UGWT has gained high attention due to its long range inspection capabilities [2], ability to perform in-situ testing (as a rapid screening tool) and reliability when compared to other defect detection methods. Recent attempts have been reported on the advancements of resolution, sensitivity and mode purity [3,4], the latter defined as obtaining a single wave type of defined motion without contamination by other waves superimposed, for example, obtaining just pure in-plane motion or out-of-plane motion. Through improvements to the transducer and enhancements to the level of mode purity, the resolution of the system and therefore capability of flaw detection is increased. Much of the improvements have been in the field of digital signal processing and real time analysis, with arrays of transducers providing input, hence improvements in transducer geometry for accuracy and mode purity have the potential for exploitation.

The three potential modes of vibration utilized in testing plate-like structures (for long range UGWT where the operating frequency range tends to be from approximately 20 to 100 kHz) are A0, S0 and SH0 [5,6]. Whilst an infinite number of higher order modes are present, the commercial inspection interest is towards increased purity of the shear horizontal mode, SH0, due to its non-dispersive nature and its isolated excitation [7,8,9,10,11,12].

Transducers with materials comprising Lead Zirconate Titanate (PZT) and Electromagnetic Acoustic Transducers (EMAT) are the commonly chosen means of exciting structures for UGWT applications [13,14]. The state-of the-art dry coupled surface shear piezoelectric elements were developed as a result of work performed by Alleyne and Cawley [15,16] on this subject. This particular design was then further developed by Elborn [17], where an alumina layer was inserted to protect the piezoelectric transducer to avoid the direct contact with the pipe and the connection to the piezoelectric transducer was obtained through a wrapped around electrode [18]. However, the design methodology was carried out assuming an ideal transducer behaviour, with exclusively empirical assessments of the design quality and performance.

The numerical analysis of mode excitation in rods and plate-like structures has shown inconsistencies between the numerical and the experimental results [19,20,21,22]. The state-of the-art dry coupled surface shear piezoelectric designed by [17] has been validated, both numerically and experimentally, in rod-like structures, showing an unexpected flexural mode, interpreted as reflection from the waveguide, thus making the signal interpretation more challenging in relation to the objective of inspection [20,21]. Nonetheless, the explanation of the authors was based on mere signal interpretation, lacking a detailed and systematic analysis on anomalies in transducer behaviour.

The findings in [22] further strengthen the evidence for the aforementioned anomalies, showing discrepancies between the numerically simulated SH0 mode purity and experimental results in plate-like structures. Further observations by [22] include the radiation of an A0 mode along the preferential direction of SH0. While the scope of work was not to investigate singular transducer behaviour, the author failed to provide an explanation of the physical cause of those inconsistencies.

The anomalies might be attributed to the peculiar design of the transducer, as it features a wrap-around electrode configuration. Recent work on transducers with this characteristic has shown variations in the frequency response function when compared to a non-wrapped around case [23,24]. To the best of the authors’ knowledge, the influence of this design choice on transducer behaviour has not been quantified in literature yet. Moreover, a correlation between transducer’s design based on [17] and mode purity of SH0 is still missing. This paper provides an investigation of the effect the wrap around electrode causes to the functional performance of the transducer.

In this paper we present: the characteristics of the current state-of-the-art thickness-shear transducer and its experimental characterization, with a detailed presentation of inconsistencies between theoretical and experimental behaviour; the numerical investigation of the inconsistencies in the ultrasonic output, along with their physical interpretation; finally, novel design guidelines, based on numerical analysis, aimed at improving SH0 mode purity, along with their experimental validation on a working prototype.

## 2. Analysis of a Current, State-of-the-Art, Generic, UGTW, Transducer, Design

### 2.1. Design of the Transducers

**Figure 1:**
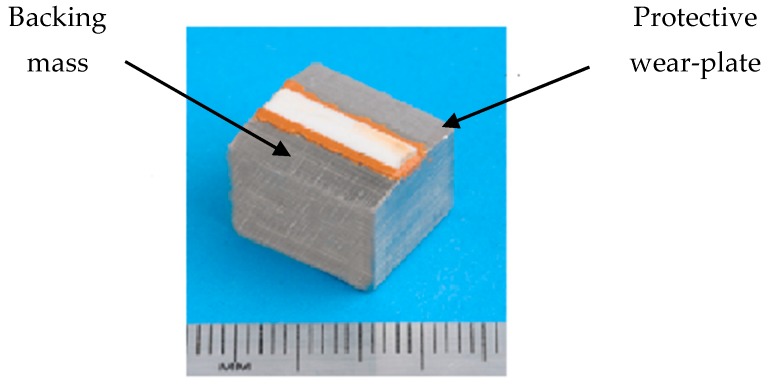
Current state-of-the art transducer typical of those found with wrapped around electrodes. Labels are inserted to indicate the two features visible externally, the backing mass and the protective wear-plate.

The transducer is composed of three main components, a piezoelectric element (PZT), a protective layer (wear plate) and a backing solid block, as shown in Figure 1. Such a configuration in the remainder of the paper is defined as configuration A (where the electrode is wrapped back on the PZT element). The piezoelectric element is a soft ceramic PIC 255 vibrating 1–5 thickness-shear mode [25]. As a thickness-shear element, the imposition of an electric field normal to the polarization axis induces a shear stress to the specimen and the material is polarized along its length [26].

**Figure 2:**
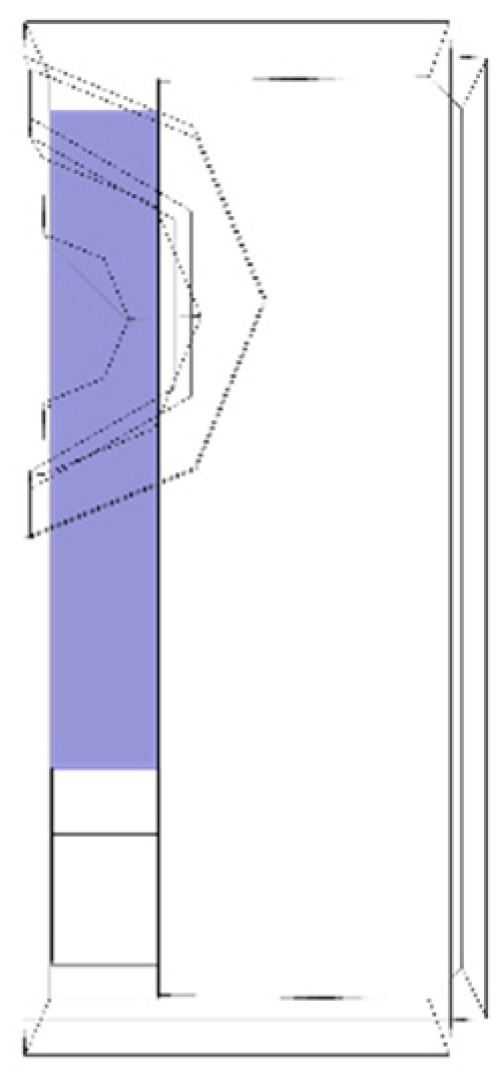
Imported CAD of the transducer viewed from top. Due to symmetry along the longitudinal axis only half the transducer is shown. Backing mass is in mechanical and electrical contact with the piezoelectric element. The actuation area on the top electrode of the piezoelectric element is highlighted in blue (it doesn’t extend to the full length of the transducer due to the wrapped around electrode, configuration A). The access cable is cleared from internal electrical connection.

The length of the piezoelectric element is 13 mm for this study. The applied voltage to the element is applied through electrodes placed on either surface configurations, which also necessitates a study cable connection and access hole in the solid mass. The imported drawing of the transducer is shown in Figure 2. The electrical contact to the piezoelectric element is provided through plated electrodes which are presently wrapped around from the bottom surface to the top. Due to this wrapped around system, the actual excited length of the piezoelectric element is only 10 mm (shown in blue in Figure 2) but a discontinuity in voltage potential is created around the wraparound termination.

To prevent any mechanical failure due to the brittleness of the piezoelectric ceramic (and due to the dry coupling to the workpiece during inspection), the piezo-ceramic is bonded to an alumina layer which acts as a wear plate maintaining a direct contact with the surface of the waveguide, with in-plane dimensions exactly corresponding to those of the piezoelectric element.

The third element is a (near) cuboid of stainless steel of length 13 mm and height 13 mm, which increases the flexural stiffness of the system and provides an appropriate distribution of pressure to the piezoelectric element when it is preloaded on its upper surface. The use of such a backing mass makes also the device portable, easy to use and capable to resist to test in harsh environments: an asymmetry is present in the backing mass, due to the presence of a hole providing wiring connection to the piezoelectric element.

### 2.2. Experimental Setup

**Figure 3:**
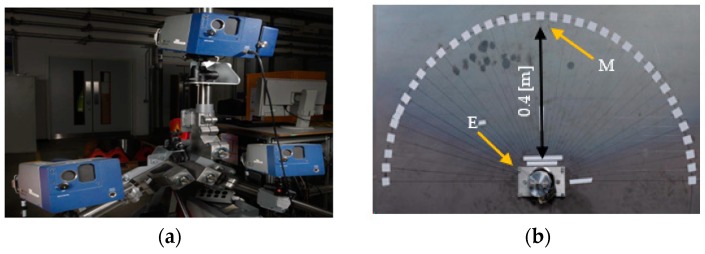
Laboratory experiments to characterize the transducers, 3D Laser Doppler Vibrometer used to monitor the surface vibration at the point of interest (**a**) PSV-400 3D Doppler Vibrometer used to monitor the surface vibration at the point of interest (**b**) experimental setup illustrating the point of excitation (labelled as E) and monitoring point (labelled as M) and the corresponding distance in meters.

In this section, the mode purity of a shear horizontal mode is assessed for Configuration A: the displacement is measured on a test sample using a 3D scanning Laser Doppler Vibrometer (3D-LDV) [25], following established practice in the literature, see [20,22,27]. The Polytec 3D-LDV (PSV-400-3D-M) shown in Figure 3a measured the surface displacement at the monitoring point M as illustrated in Figure 3b [25]. The point M was at distance 0.4 m from the exciting transducer, defined as an emission point E. The point M was selected as it presents the theoretical highest directivity of SH0 [22].

The mild steel plate chosen for validation was a square plate of dimension 2.0 × 2.0 × 0.003 m. 

The transducer was placed in the centre of the plate with a loading device to control the preload force, applied as a static out-of-plane load [19]. The device was excited with a 5 cycles Hann windowed burst with a centre frequency of 90 kHz, achieving an appropriate mode separation in a limited space for this analysis. 

The software Disperse was used to calculate the time of arrival (*ToA*) for the potential propagation modes include S0, A0 and SH0 [28]. The *ToA* was calculated according to the following equation [20]

(1) $$ToA=\frac{x}{V_{gr}}$$

where *x* is the distance and *V_gr_* is the group velocity of the mode of interest. The *ToA* of the three modes is indicated in Table 1.

Such an input signal was validated as adequate in the literature [20]. Due to the minimal variance of the frequency response function in the range 30–90 kHz [13], selecting only one excitation frequency is acceptable to evaluate the behaviour of the transducer and the general response. The transducer was excited using the commercially available Teletest Focus+ [2].

### 2.3. Experimental Results

**Figure 4:**
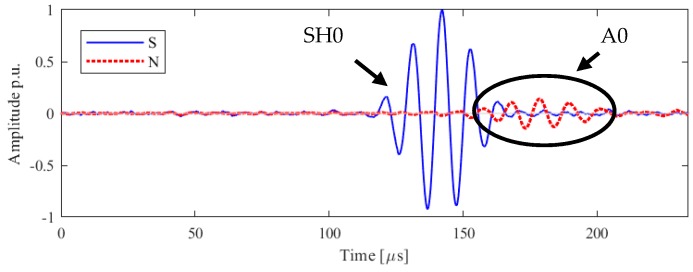
Laboratory experiments of the in-plane (blue), ‘S’, and out-of-plane, ‘N’, (red) velocities along the line orthogonal to the axis of vibration, for Configuration A. Normalized data are shown: modes are identified and labelled accordingly.

The excitation directivity of interest in the scope of this study is the axis perpendicular to the direction of vibration, which corresponds to the direction of propagation of the SH0 mode, as expected from the results in the literature [20]. In Figure 4 the in-plane and out-of-plane displacement are plotted as function of the time of arrival at the point M: since the interest is in the in-plane vibration, the in-plane amplitude is referred throughout the article as ‘S’, signal and the out-of-plane amplitude is referred as ‘N’, noise. The amplitude is normalized to the in-peak amplitude of the shear horizontal mode.

The experimental result for the receiving point perpendicular to the axis of vibration is illustrated with a SH0 mode arrival time of 121 μs, which is of high amplitude and quality with a low signal to noise ratio, thus can be used for inspection. However, another unexpected mode with predominantly out-of-plane components appears at 153 μs: due to the nature of motion and of the time of arrival, it can be identified as the A0 mode. This spurious wave mode must be reduced or removed to obtain high mode purity. Whilst the existing transducer provides complete functionality, for future improvement and ease the signal interpretation mode purity of SH0 needs to be further studied.

To the best of the authors’ knowledge, this phenomenon has never been reported in the literature. It can be inferred that the transducer is not moving only along the axis of vibration, as expected by the thickness-shear movement of the piezoelectric element, but the full assembly of the transducer is generating an unexpected mode which could result in a detriment in the mode purity of SH0. In the following section, a numerical model of the full assembly will be compared with the experimental results and the insight of the simulation will be deployed to test this hypothesis. It will be shown, as a novel finding, that the amplitude of the spurious A0 mode is due in part to the wrap around electrode.

## 3. Numerical Analysis of the Transient Behaviour for Configuration A

### 3.1. Background of the Model

The wrap around electrode is present as it solves manufacturing constraints, which would need to be resolved before testing new designs. Therefore, validated numerical models will be used to demonstrate the potential of further design changes.

The finite element method (FEM) was used to evaluate the guided wave propagation of a thickness-shear piezoelectric transducer and its complete assembly as a 3D geometric transducer. Such a method has shown excellent agreement against experimental results, and a physical insight into the characteristic of the transducer is readily available [20,21]. The modelling was carried out using Comsol Multiphysics [28], which is currently used for ultrasonic transducers and guided waves studies [29,30,31,32]. The experimental analysis was replicated numerically, to predict the time of arrival, to study the purity of the latter and compare the output with a simple point source model.

**Figure 5:**
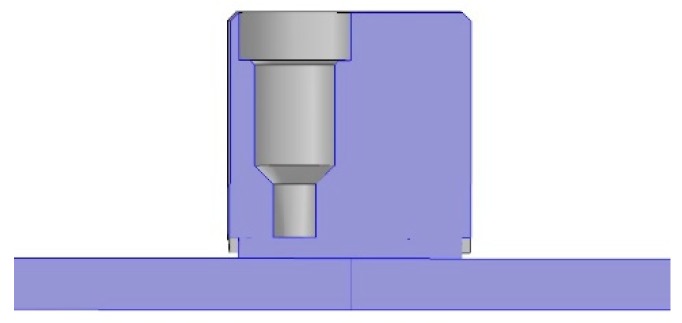
Picture showing the symmetry condition imposed on the transducer and on the plate, highlighted in blue.

The transducer was placed on the centre of the steel plate. Since the transducer and the plate are symmetrical along the longitudinal axis of vibration, a symmetry condition was imposed on the transducer and on the plate to reduce the computational effort. The symmetry condition is highlighted in Figure 5. To reproduce the experimental conditions, radius and thickness of the plate were set as 0.4 m and 3 mm respectively.

As far as the interaction between the alumina wear-plate and the waveguide, it was assumed that surface of contact is completely flat. Thus, the two surfaces should not move relative to each other, an assumption which is generally valid as the whole purpose of the static vertical loading is to eliminate stick slipping on the wear to workpiece contact.

Material properties are used assuming linear elastic behaviour of the system: as far as the piezoelectric element is considered, only the mechanical properties are considered. This assumption has been proved valid in previous numerical finite element modelling of the transducer [19,20,21]. Material properties are reported in Table 2.

**Figure 6:**
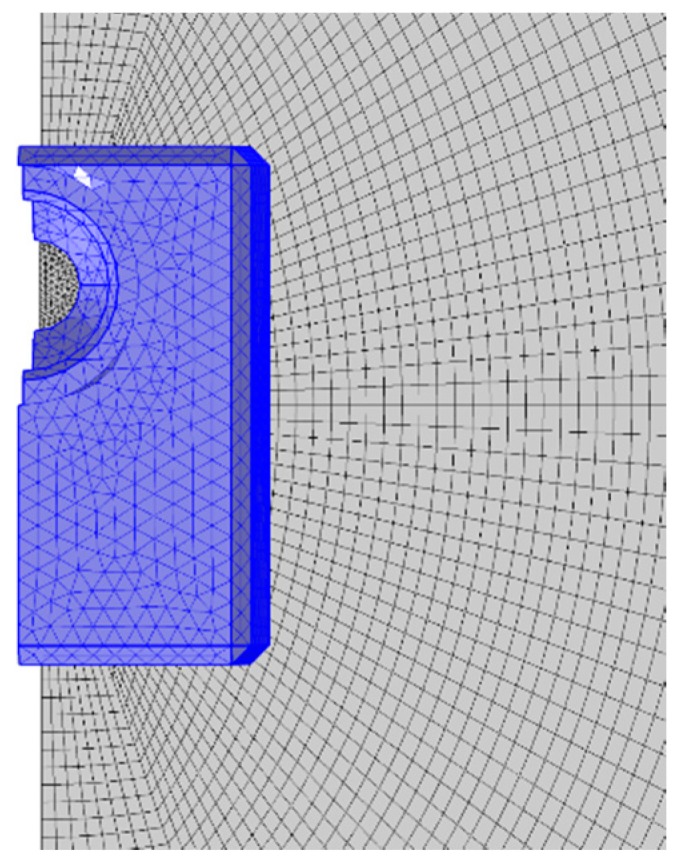
Picture showing the tetrahedral mesh on the transducer (highlighted in blue) and the quadratic mesh on the plate.

**Figure 7:**
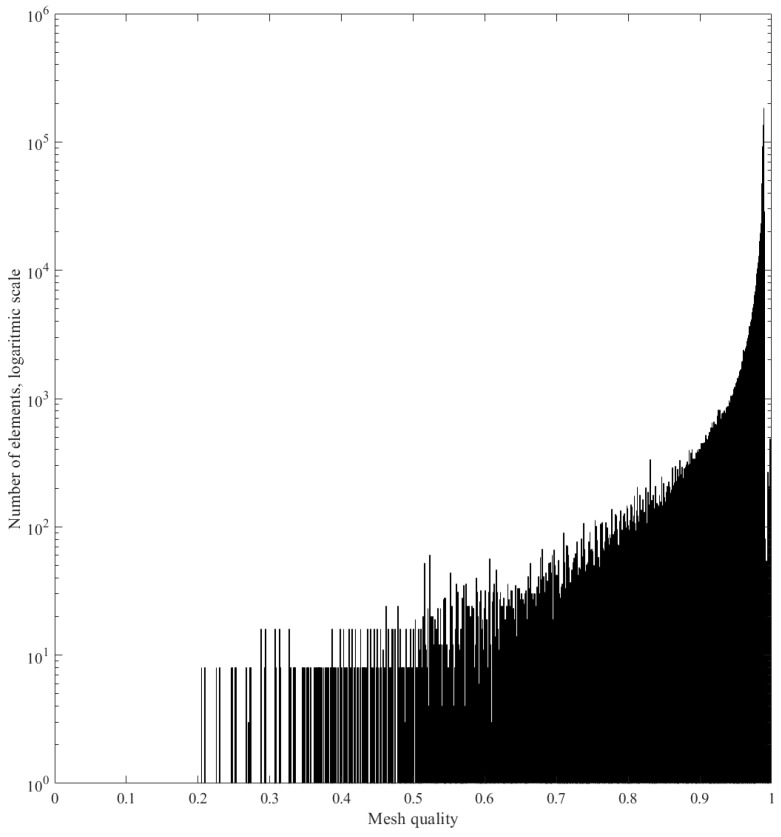
Plot of the number of elements as a function of mesh quality. Note that the ordinate axis is in logarithmic scale.

The transducer was excited with an in-plane surface load (along the length of the device) and the signal was a 5 cycles Hann-windowed burst at 90 kHz (the same as in the experimental investigation). The numerical analysis was carried out using the solid mechanics module for computational efficiency. The mesh size of the waveguide in the model was computed according to the following equation,

(2) $$h=\frac{c}{N\times f0}$$

where *c* is the velocity of the slowest mode, N is the number of cycles and *f*0 is the frequency of interest: eight elements were used represent the wavelength of the slowest mode, the mesh size h was calculated as 3.6 mm. Quadratic elements were used to mesh the plate: such resolution of the mesh size was previously validated to be adequate in the literature [20]. Due due to the irregularity linearity of the transducer geometry, tetrahedral elements with a reduced order of magnitude were used to mesh the assembly (0.36 mm): thus, the transducer’s behaviour could be modelled more accurately. The mesh for the assembly and the waveguide are shown in Figure 6: moreover, a histogram with the number of elements as a function of mesh quality indicating that the meshing procedure is appropriate is presented in Figure 7.

### 3.2. Numerical Results

**Figure 8:**
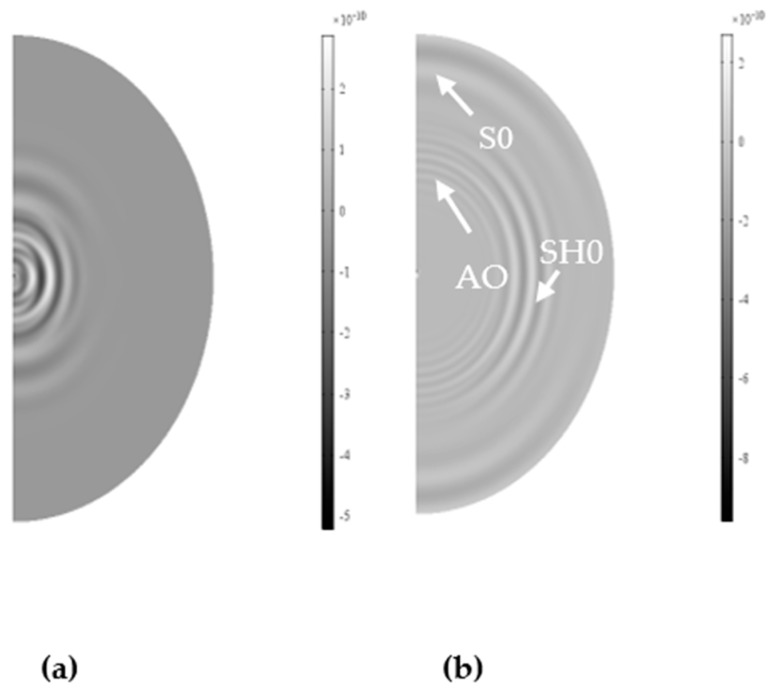
Surface in-plane displacement plot at 50 µs (**a**) on right; at 100 µs (**b**) on left for Configuration A. Modes are identified and labeled accordingly.

In Figure 8a, the in-plane displacement at 50 μs is presented. The symmetric Lamb mode, S0, is propagating along the axis of vibration while the shear horizontal mode is propagating on the orthogonal axis, as theoretically expected. Moreover, the shear horizontal wave is showing a higher intensity than the S0, is well defined and of high relative amplitude. The surface plot at 100 μs is also presented in Figure 8b. The S0 mode has reached the edge, while the SH0 is still propagating, with the separation between modes making clear the presence of the A0 mode along the longitudinal axis of vibration. The in-plane and out-of-plane component of displacement were also extracted at the defined receiver M on the border of the plate.

**Figure 9:**
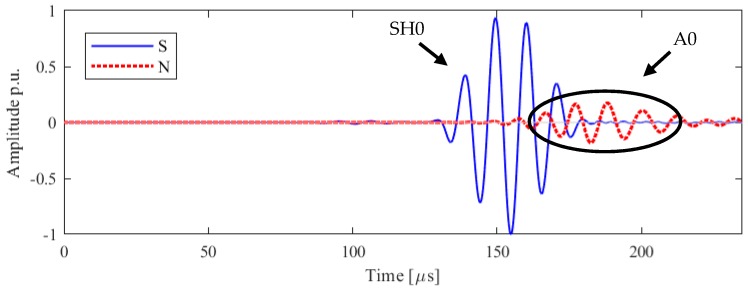
Configuration A numerical results of the in-plane (blue), ‘S’, and out-of-plane, ‘N’, (red) velocities along the line orthogonal to the line of vibration. Normalized data are shown: modes are identified and labelled accordingly.

The two components of displacement are plotted for configuration A, with the wrap around electrode, the receiving point in Figure 9 where only the SH0 mode is expected. It is confirmed that the time of arrival of 125 μs is consistent with the theoretical and experimental calculation [20]. As expected, the shear horizontal mode presents only an in-plane vibration: however, a spurious mode is appearing after the desired mode. This second mode is mainly out of plane and arriving at 153 μs, identified as A0.

**Figure 10:**
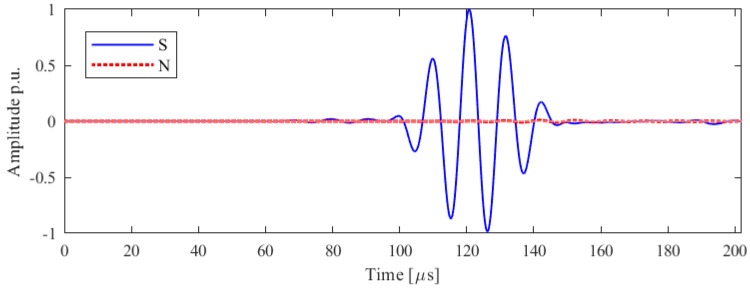
Point source numerical results of the in-plane (blue), ‘S’, and out-of-plane, ‘N’, (red) velocities along the line orthogonal to the line of vibration. Normalized data are shown.

The model then agrees with the experimental findings and show that the introduction of the real design of the transducer is fundamental to assess the mode purity of SH0 and in general the behaviour of the transducer. The transducer in this configuration is generating an omnidirectional A0 not desired and it is attributed to the electrode lay-out and the size of the transducer. As mentioned in the introduction, currently arrays of thickness-shear transducers are designed assuming the transducers vibrate uniformly, henceforth the generated wave-front can be considered as generated by the linear superposition of point sources. In Figure 10 the output along the perpendicular direction as excited by a point-source is plotted.

The point-source case represents the ideal case and the benchmark for inspection purposes, since only the SH0 mode is generated. However, such a model fails to predict the current ultrasonic output of configuration A. Thus, in the next section, the numerical model is further analysed to evaluate how the extension of the surface load and the diminution of the non-excited area can modify the out-of-plane motion.

### 3.3. Physical Interpretation of the Pattern of the Transducer

**Figure 11:**
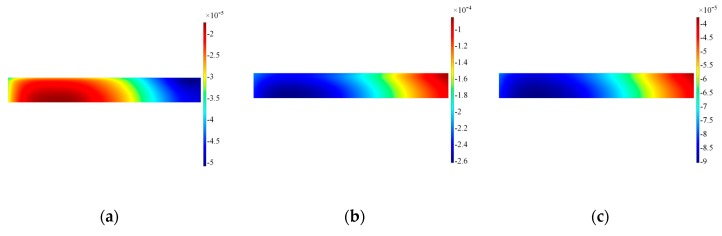
Surface plot for in-plane velocity on the alumina wear-plate, Configuration A. Note that data are plotted at timeframes of 100 μs (**a**), 300 μs (**b**) and 500 μs (**c**).

**Figure 12:**
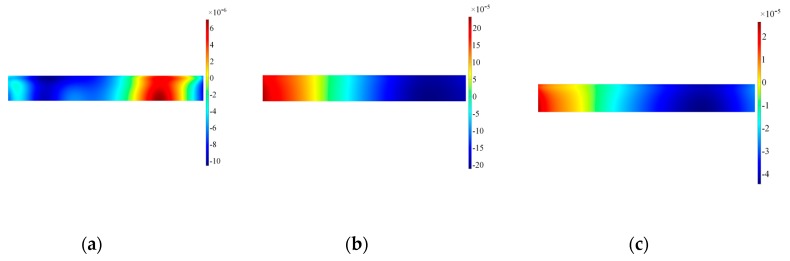
Surface plot for out-of-plane velocity on the alumina wear-plate, Configuration A. Note that data are plotted at timeframes of 100 μs (**a**), 300 μs (**b**) and 500 μs (**c**).

The numerical results have confirmed the experimental findings concerning an unexpected A0 mode compromising the mode purity of SH0. It is then of paramount importance to investigate the features contributing to the excitation of this spurious mode. The first feature evaluated is the interface transducer-waveguide, since it is the vibration along this boundary ensuring the excitation of guided waves inside the structure. The fundamental assumption is uniform vibration along the longitudinal axis of the transducer: thus, surface plots have been extracted at the interface both for in-plane and out-of-plane displacement to verify the assumption, as shown in Figure 11 and Figure 12. Three different time steps have been selected to ensure the transient evolution is fully appreciated.

The results for the in-plane velocity in Figure 12 indicate clearly how the vibration is not evenly distributed across the length of the transducer and the area on the left border where the wrap around is located presents remarkable difference with the remainder of the transducer. Furthermore, distribution of the in-plane velocity is concentrated more prominently where the access cable is located. The corresponding results for the out-of-plane velocity are shown in Figure 12. The distribution of positive and negative out-of-plane velocity along the length prove that the transducer is bouncing up and down at the edges, thus the main in-plane vibration is disturbed by the rotation of the transducer. Note also that the modulus of the in-plane and out-of-plane velocities are in the same order of magnitude, increasing in time for the out-of-plane: therefore, when the driving signal diminishes in time the inertia of the sensor increases its effect on the transducer.

**Figure 13:**
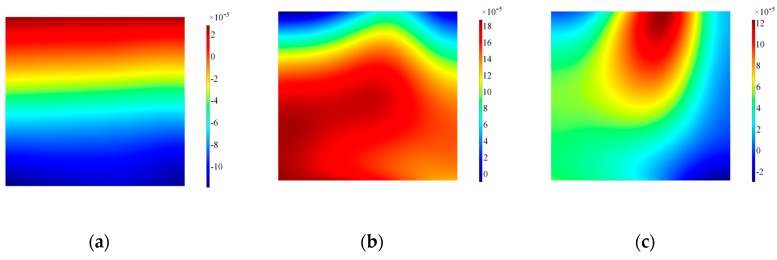
Surface plot for the in-plane velocity on the side of the backing mass, Configuration A. Note that data are plotted at timeframes of 100 μs (**a**), 300 μs (**b**) and 500 μs (**c**).

**Figure 14:**
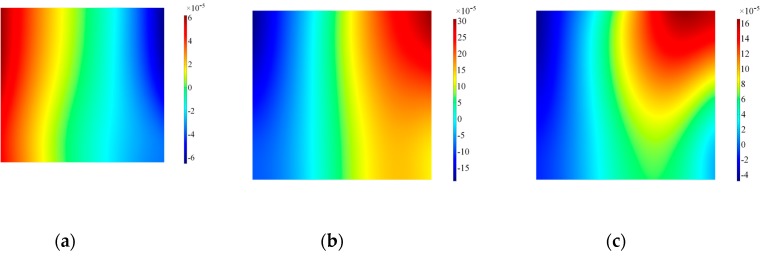
Surface plot for the out-of-plane velocity on the side of the backing mass, Configuration A. Note that data are plotted at timeframes of 100 μs (**a**), 300 μs (**b**) and 500 μs (**c**).

To highlight the effect of the real design of the transducer, similar time frames were also extracted as surface plots on the lateral surface of the backing mass. The results for the backing mass plotted in Figure 13 indicate that at the beginning the motion imposed by the piezoelectric element is effectively a thickness-shear motion, with a higher modulus on the bottom surface of the backing mass. As the excitation time progresses the distribution of velocity becomes more chaotic and difficult to interpret: both in-plane and out-of-plane velocities are influenced by the presence of the hole, and the backing mass experiences a rotation as shown by the out-of-plane in Figure 14.

Thus, the finite element analysis for configuration A shows that three main elements are influencing the ultrasonic output of the transducer, i.e., the electrical lay-out, the position of the access cable and the size of the sensor. Since the first parameter seems to be more influencing the in-plane vibration and the spurious A0 mode, in the remainder of the section only the actuation length of the transducer will be modified and defined as configuration A’.

### 3.4. Numerical Results on a 3D Geometric Transducer without a Wraparound Electrode (Configuration A’)

In Section 2, the actuation area of the generic piezoelectric transducer was described as 10 mm, corresponding to the positive side of the electrode, which is receiving the exciting signal. The negative electrode is wrapped around with a spacing of 3 mm on the upper surface to provide insulation against a short circuit. Using the validated numerical finite element model, a prediction of the ultrasonic output is computed when the actuation length is modified from 10 to 13 mm in two parallel continuous strips, but the access hole is kept to study the influence of the wrap around electrode (Configuration A’).

**Figure 15:**
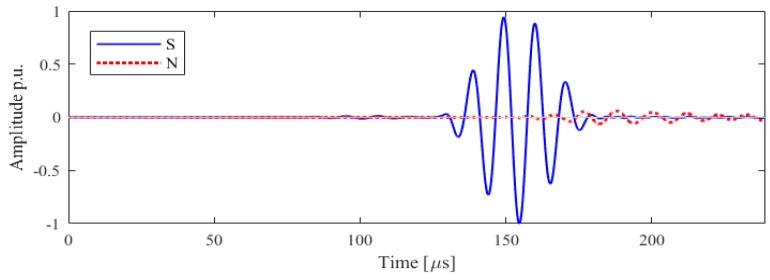
Configuration A’ results of the in-plane (blue), ‘S’, and out-of-plane, ‘N’, (red) velocities along the line orthogonal to the line of vibration. Normalized data are shown.

In-plane and out-of-plane displacement at the point perpendicular to the axis of vibration for the bespoke transducer design is illustrated in Figure 15. SH0 mode is excited in high purity at the direction perpendicular to the axis of vibration while the unexpected A0 mode has an amplitude which is more than halved, a considerable benefit in terms of mode purity. Thus, the actuation length and the position of electrode are shown to make a considerable difference in terms of the out-of-plane motion and improve the transducer performance. However, results in Figure 15 show that there is still a small trail of signal attributable to A0 even when a more ideal source of excitation is considered: thus, Configuration A’ on its own would not be sufficient to assure a pure SH0 mode.

**Figure 16:**
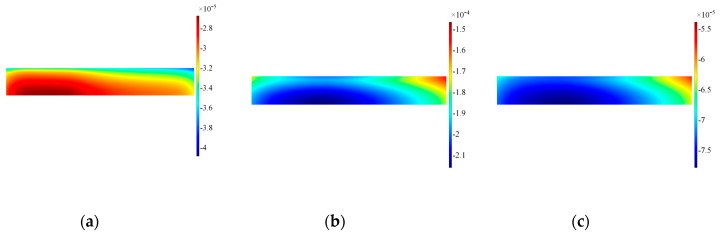
Surface plot for in-of-plane velocity on the alumina wear-plate, Configuration A’. Note that data are plotted at timeframes of 100 μs (**a**), 300 μs (**b**) and 500 μs (**c**).

**Figure 17:**
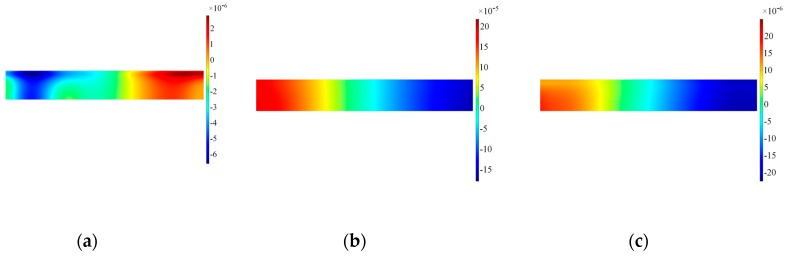
Surface plot for out-of-plane velocity on the alumina wear-plate, Configuration A’. Note that data are plotted at timeframes of 100 μs (**a**), 300 μs (**b**) and 500 μs (**c**).

**Figure 18:**
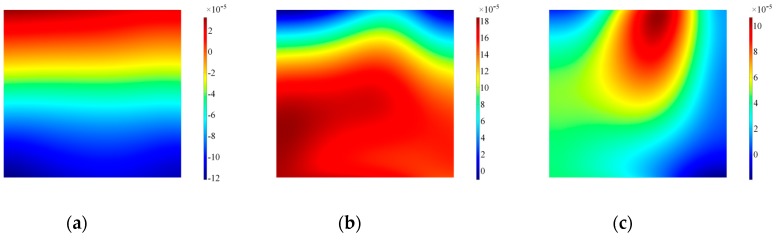
Surface plot for the in-plane velocity on the side of the backing mass, Configuration A’. Note that data are plotted at timeframes of 100 μs (**a**), 300 μs (**b**) and 500 μs (**c**).

**Figure 19:**
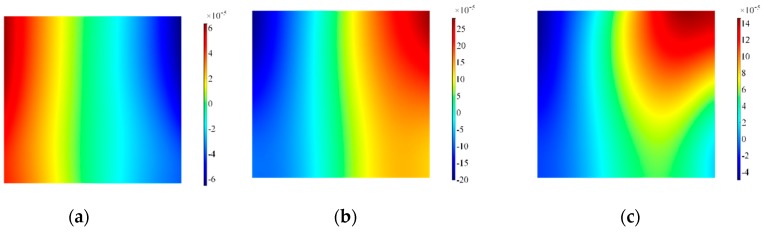
Surface plot for the out-of-plane velocity on the side of the backing mass, Configuration A’. Note that data are plotted at timeframes of 100 μs (**a**), 300 μs (**b**) and 500 μs (**c**).

The results at the interface transducer waveguide for in-plane velocities are plotted in Figure 16: clearly the distribution of in-plane velocity becomes more even across the length, while the out-of-plane component is more confined to the corner in comparison to Configuration A, as shown in Figure 17. The surface plots for the backing mass in Figure 18 and Figure 19 shows again that the hole influences the vibration pattern of the transducer, especially in the later time of excitation.

As explained in the previous section the access cable creates an asymmetry in the centre of mass, henceforth a moment on the backing block, which would then lead to distortion in the vibration pattern. Therefore, the construction of the prototype had to be carried out eliminating the access hole of the cable and ensuring electrical contacts on the sides of the transducer.

## 4. Development and Testing of a New Configuration

### Numerical Results on a 3D Geometric Transducer Without a Wraparound Electrode and with Geometry Modified (Configuration B)

Numerical and experimental findings have indicated that Configuration A presents an undesired pattern of vibration compromising the mode purity of SH0. Thus, it is of interest to develop a new transducer with similar geometrical dimensions but able to meliorate the objective of the device. Thus, a new configuration named B has been designed, developed and tested both numerically and experimentally: such a configuration has undergone some practical modification, since the electrical layout of the transducer has been removed and substituted by a continuous electrode configuration. The access cable could then also be removed. The main difference in the system is in the electrical connectivity, since the positive electrode is obtained by connecting the upper surface of the transducer with an external cable soldered to the backing block: the negative side of the circuit is closed through a cable soldered on a layer of copper tape. The copper tape is inserted between the interface piezoelectric element- wear-plate. Table 3 outlines the main constructive differences, Y and N standing for the positive or negative presences of the indicated features.

Note that the insertion of the copper tape and the connection through the backing mass had as a drawback the possible reduction of the amplitude, since connection would lose the practicality of the wrap-around system: however, scope of the prototype is to demonstrate the enhancements of SHO mode purity due to modification of geometry and electrical lay-out, henceforth loss of amplitude is at this stage acceptable.

**Figure 20:**
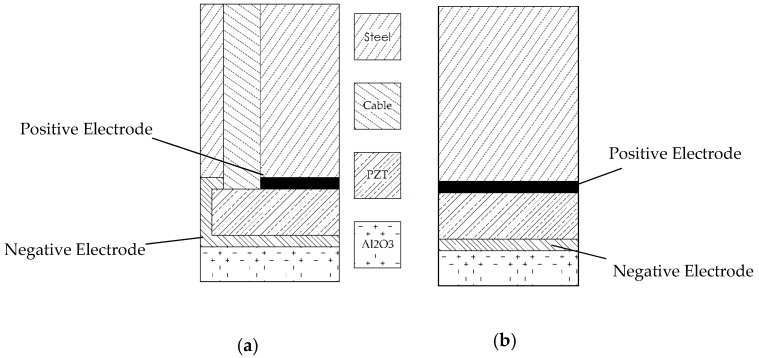
Transducer (**a**) Configuration A (wrap around electrodes) and (**b**) Configuration B (proposed modified electrode attachment and geometry). Note that configuration A presents an access hole for the electrical connectivity: positive electrode is presented with a thicker black line and negative electrode along the alumina layer. The representation is exaggerated to help the understanding.

A schematic representation of the differences between the two configurations is shown in Figure 20.

**Figure 21:**
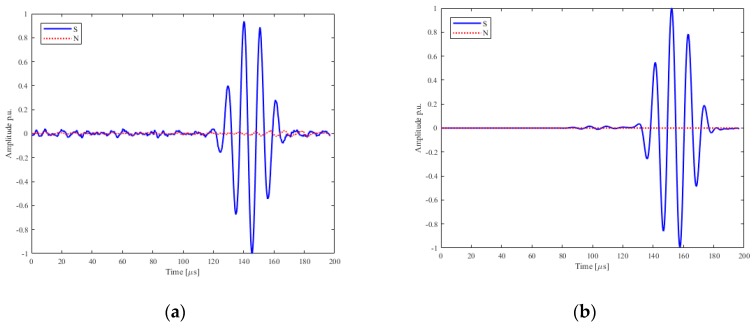
Configuration B results of the in-plane (blue), ‘S’, and out-of-plane, ‘N’, (red) velocities, experimental (**a**) and numerical (**b**) along the line orthogonal to the line of vibration. Amplitude and normalized data are shown.

Experimental and numerical analysis were carried out according to the procedures described in Section 2 and Section 3. The experimental and numerical results for the shear horizontal mode are shown in Figure 21a,b.

It is evident that the SH0 mode has been excited with higher mode purity on the direction of interest, and no spurious mode was evident: thus, the transducer along the orthogonal direction follows the idealized pattern of the in-plane point source, and it can be assumed that the surface of the transducer is vibrating uniformly along the direction of vibration. Any existing out-of-plane vibration would then be buried under the noise-level, therefore the interpretation of received signals from defects would be improved if the Configuration B is considered.

It has also been proven that both excitation area of the transducer and geometry of the backing mass influences the excitation of undesired modes: both those factors should then be taken into consideration for an industrial development of the prototype. The diminution of the amplitude indicated by the experimental results need further investigation in the joining technology between the elements constituting the transducer.

## 5. Conclusions

The full assembly of a generic thickness-shear transducer has been modelled with finite element analysis to characterize its ultrasonic output for plate-like structures. The model was validated against work present in the literature and against an experimental validation with the 3D scanning laser Doppler Vibrometer. The results for the Lamb mode have shown no inconsistencies with previous results and with the experimental validation. However, both the numerical and experimental validation has shown that on the direction perpendicular to the axis of vibration has an out-of-plane component which corresponds to the A0 mode. Such mode is unexpected and decrease signal to noise ratio of an inspection and complicates the signal interpretation and processing. Moreover, it has been shown that the transducer is not only vibrating in plane, but an out-of-plane vibration is also present, which is generating an omnidirectional A0 mode. Such a mode does not appear in the point source model, which then should be discarded as approximation, since it fails to evaluate the mode purity of the shear horizontal mode.

An experimental and numerical study was conducted to reduce this effect and it has been proposed to remove the wrap-around electrode exciting the piezoelectric element and to extend the actuation length from 10 to 13 mm. This proposed change followed some indications in the literature which suggests inconsistencies in the vibration pattern when a wrapped around electrode is present [19,20]. The Configuration A has then been modified and at first studying numerically (Configuration A’), showing a potential improvement exists when the actuation length is increased. The indication from the numerical simulation have then indicated the possible areas for improvement, exploited to create a prototype (Configuration B): the promising result the prototype can then be further used as a benchmark to optimize arrays of transducers, miniaturize the transducers as suggested by Marques [22] and study the effect of the coupling force on a more regular geometry.

## Figures and Tables

**Table 1:** *V_gr_* and *ToA* of the three potential modes.

Mode	*V_gr_* [m/ms]	*ToA* [μs]
S0	5.4	74
A0	2.6	153
SH0	3.2	125

**Table 2:** Material properties used for FEA modelling.

Properties	Unit	Waveguide	Transducer	Block	Wear-Plate	Adhesive
Density	[kg/m^3^]	7800	7800	8030	3960	752
Young’s modulus	[Pa]	207 × 10^9^	110 × 10^9^	193 × 10^9^	370 × 10^9^	1.47 × 10^9^
Poisson’s ratio		0.3	0.36	0.25	0.22	0.4082

**Table 3:** Different features of the assembly for Configuration A and B.

Characteristics	A	B
Cable	Y	N
Wrap El.	Y	N
Continuous El.	N	Y
Copper tape	N	Y
